# Activity Budget Comparisons Using Long-Term Observations of a Group of Bottlenose Dolphins (*Tursiops truncatus*) under Human Care: Implications for Animal Welfare

**DOI:** 10.3390/ani11072107

**Published:** 2021-07-15

**Authors:** Tim Huettner, Sandra Dollhaeupl, Ralph Simon, Katrin Baumgartner, Lorenzo von Fersen

**Affiliations:** 1 Nuremberg Zoo, Am Tiergarten 30, 90480 Nuremberg, Germany; sandyerl@aol.com (S.D.); ralph.simon@stadt.nuernberg.de (R.S.); katrin.baumgartner@stadt.nuernberg.de (K.B.); 2 Institute for Biosciences, University of Rostock, Albert-Einstein-Strasse 3, 18059 Rostock, Germany; 3 Zoo Duisburg, 47058 Duisburg, Germany; 4 CoSys-Lab, Antwerp University, 2020 Antwerp, Belgium

**Keywords:** animal welfare, zoo animal behavior, bottlenose dolphin, dolphin behavior, activity budgets, keeper ratings

## Abstract

**Simple Summary:**

Behavioral observations are widely considered easy-to-apply and straightforward animal welfare measures for animals under human care. In the present behavioral study, the activity budgets of a group of bottlenose dolphins are analyzed for nine different observation periods over five years. For some of the time periods, some extraordinary events took place, such as construction work. Our results show that activity budgets are significantly impacted by individual factors (e.g., age) and external factors (e.g., group composition). Furthermore, the presence of construction noise significantly affects the occurrence of other behaviors (fast swimming), as well as the dolphins’ performance during daily training sessions. We find that behavioral monitoring is an essential tool for assessing and ascertaining how the welfare of dolphins under human care can be improved, especially if used together with other measures, such as keeper ratings or health assessments.

**Abstract:**

Zoos and aquaria must provide optimal husbandry conditions and the highest welfare standards for their animals. How the welfare state of an animal or a group of animals can be precisely assessed is currently under debate, and new approaches are necessary to reliably evaluate changes in welfare. One particular measure that is easily applicable is behavioral observations. However, for dolphins and other cetaceans under human care, reliable behavior-based measures are rare. Using long-term observations of a group of bottlenose dolphins, we investigate how their activity budgets and different behaviors changed over time and are impacted by construction noise. Additionally, we investigate whether behavioral differences are also reflected in changes in the dolphins’ performance during daily training sessions. Our results show that construction noise significantly alters the dolphins’ behavior. Play behavior decreases during construction periods; most importantly, the frequency of fast swimming activities significantly increased, and at the same time, a decrease in training performance is found. Additionally, inter- and intraindividual behavioral differences are attributed to factors, such as age or weaning. Significant changes in a dolphin’s activity budget can also pose potential welfare concerns. Thus, this study highlights the importance of regularly assessing and analyzing the behavior of dolphins under human care. Behavioral observations are essential welfare indicators and can—when complemented with other measures, such as assessment of training performance—provide zoo staff with important information about each individual’s state of welfare.

## 1. Introduction

Securing animal welfare has been a central aspect of animal husbandries for decades and was initiated by the publication of the Five Freedoms [1]. Some years later, Mellor and Reid’s [2] presented a first formulation of the Five Domains that build a valid framework for the broad assessment of animal welfare. Both approaches contained very similar elements, but while the Five Freedoms focused primarily on the prevention of negative experiences for the animals, the Five Domains framework focused on the animal’s mental state and recognized that welfare can be both positive and negative. The Five Domains contains the following domains: Nutrition, environment, health, behavior, and mental state. The first four domains can be defined as physical domains, whereas the last one, the mental domain, reflects psychological wellbeing. The Five Domains framework acted as a foundation for fundamental animal welfare considerations. In the most recent update, specific guidance on evaluating the negative and/or positive impacts of human behavior on animal welfare, was added [3].

Zoos play a key role in advancing the field of animal welfare as it is of paramount importance for them to create environments and husbandry conditions that ensure that the animals under human care do not suffer and are in a good welfare state. Zoos are important drivers as they can develop evaluation tools and test them for validity. One important step was to find and define indicators that were directly measurable. In this regard, physiological indicators play a very important role. High activity of the adrenal cortex is part of life, and thus, not necessarily bad. It can be estimated by measuring, for example, the glucocorticoid cortisol. Though animals have evolved mechanisms to cope with short-term stressors, long-lasting exposure to stress can result in a situation where the biological cost of stress is too high. In this case, animals can succumb to disease or fail to reproduce or develop properly [4,5,6,7,8]. However, there are cases where cortisol alone or other hormones do not help to differentiate between non-threatening stress and distress [9], and thus, physiological indicators should not be used solely to assess the welfare of animals [10]. More importantly, it means that other parameters should be included to evaluate the status of an individual. While indicators like breeding records, life expectancy, or the prevalence of diseases can provide long-term information, physiological parameters can be very informative on a day-by-day assessment. However, even if hormones and other parameters are parts of the measures that need to be included in daily welfare assessments, one of the most powerful indicators is behavior, as an animal’s welfare state can be inferred from its actions.

Especially with zoo animals, conducting behavioral studies is quite straightforward because the animals can be easily accessed and observed. However, there are also limitations—the most important is the workload for the observers. In behavioral studies, the activities of the animals are recorded throughout a monitoring period, and the amount of time spent performing each activity provides a spectrum of behavior, which can be used to differentiate individuals who are behaving abnormally or even indicate if they are experiencing stress. In many of these studies, behaviors are measured by using an activity budget, which is the percentage of time that the animal spends in a specific behavioral state. Activity budgets were formerly used to indirectly measure how animals met their energetic requirements [11]. However, in recent years, activity budgets were also used to investigate the extent to which the frequency and time spent in performing certain behaviors by an animal in a zoo significantly diverge from the behavior expressed in the wild [12]. Activity budget comparisons are also conducted under the premise that individual animals displaying a greater range of “wild behaviors” enjoy better welfare. However, this statement should also be taken with caution, as, for example, behavioral motivation may play a crucial role in determining the variability in behavior [13].

Further behavioral studies referred to the richness of an animal’s behavior as a positive welfare indicator. The rationale behind this is that measures that promote wellbeing in the usual way, such as enrichment, training, and complex environments, also lead to a higher behavioral diversity (see Miller et al. [14] for a review). Using the so-called behavioral diversity index [14], several studies looked at the richness of the behavior displayed in different species housed under human care, e.g., elephants [15] or lions [16], as well as in marine animals, such as fur seals [17] or bottlenose dolphins [18], and how they are affected by environmental factors.

To assess how behavior and welfare are affected by external disturbances, some particular behaviors can be used as key indicators of an animal’s welfare state. In large carnivores, the impact of external stressors on the frequency of stereotypical pacing is commonly investigated [19,20]. Other studies have looked at how resting behavior, enclosure use, aggression, and abnormal behavior are affected by potential stressors [16,21,22].

For dolphins, little is known about the behaviors that provide reliable information about the welfare state. Most behavioral studies were conducted with wild populations, whose behavior is mainly influenced by foraging, prey availability, or predator avoidance [23]. In zoos and aquaria, however, behavior is mostly influenced by other factors, such as daily husbandry routines [24,25], social parameters [26], external disturbances [27], enrichment [28,29,30], human–animal interactions [31], the presence of visitors [32], and the animals’ personalities [33,34,35,36].

It was only during the recent surge of welfare-related research that dolphin behavior was also specifically addressed in terms of animal welfare assessments. Thus, behavior-based indicators are still scarce, and only a few studies exist that have investigated which behaviors could be used as reliable welfare indicators.

An important first step was taken by Clegg et al. [37] with the presentation of a welfare assessment tool that was specifically developed for dolphins under human care, which included some behavior-based measures (e.g., the expression of socio-sexual behaviors and the absence of stereotypical behaviors). Other studies have incorporated different individual behaviors, such as affiliative behavior [38], play behavior [39], or the dolphins’ Willingness to Participate (“WtP”; [31,40]) in daily training sessions. One of the most important behaviors to be validated as a reliable indicator of positive welfare was found to be affiliative contacts [36], such as pectoral fin rubbing [41,42] or synchronous swimming [43].

Serres et al. [44] analyzed the effects of social and environmental factors on multiple behaviors, including affiliative and agonistic interactions, of different small cetaceans held under human care. It was found that during potentially more stressful conditions, such as disturbances or separation of the animals, affiliative body contacts (e.g., pectoral fin contacts, among other behaviors, decreased in bottlenose dolphins (*Tursiops truncatus)*, and East Asian finless porpoises (*Neophocoena asiaeorientalis sunameri*). Furthermore, bottlenose dolphins engaged in fewer agonistic behaviors during disturbances [44]. By comparing three different odontocete species, it was found, e.g., that solitary and social play decreased during noise events in three species of odontocetes, including bottlenose dolphins [45,46]. In another study, Themelin et al. [47] looked at how synchronous movements and proximity can be used to evaluate the quality of affiliative contacts in wild dolphins, as well as in dolphins under human care.

The objective of this study was to use behavioral analytic methods—in particular, by comparing activity budgets and some single key behaviors—to evaluate changes in behavior in response to changes in the environment or particular stressors during nine different observation periods (2016–2020). In the case of this study, a stressor was defined as, e.g., construction work with a significant noise level that changed the soundscape in the dolphins’ pool, or changes in group composition, due to the arrival of new animals or the departure of some animals to other facilities. Based on the results of previous studies, we focused predominantly on behaviors that are signs of either positive (affiliative behavior) or negative (fast swimming) welfare. As another measure, we used the trainer’s ratings for the performance of each animal in positive reinforcement training sessions. Several studies have already shown that trainers’ and caretakers’ assessments of traits related to the wellbeing of individual animals can be both reliable and valid across a variety of species [48,49,50].

## 2. Materials and Methods

### 2.1. Subjects and Study Site

This study included 11 Atlantic bottlenose dolphins (*Tursiops truncatus*) that were housed together with a group of Californian sea lions (*Zalophus californianus*) at Nuremberg Zoo, Germany (see Table 1). The enclosure consisted of six outdoor pools of various sizes and depths (called dolphin lagoon) and an indoor area (indoor dolphinarium) with a total water area of approximately 1900 m^2^. Usually, the dolphins had access to all pools during the observations. Only in the cold winter months (beginning of December to the end of March), the animals had access to only some of the outdoor pools. If the air temperature dropped below 0 °C, the dolphins were kept in the indoor dolphinarium and two adjacent outdoor pools that were covered by an air dome to create a climate-controlled environment.

The dolphins were trained five times per day using positive reinforcement, during which approximately 5–10 kg of fish and squid (capelin, herring, mackerel, sprat, and squid) were fed to them each day. Depending on the time of year, 2–5 animal presentations were carried out per day, replacing normal training. In addition, once per day, usually 4–6 times per week, one or two dolphins participated in behind-the-scenes interaction programs for groups of 4–6 visitors. The dolphins also participated in free-interaction “play” sessions with their trainers. Enrichment was provided regularly, including with toys (e.g., buoys, balls, fire hoses, or gym mats), ice cubes, air bubble curtains, or food enrichment.

Because of group management decisions, veterinary purposes, and population management reasons, the social grouping changed several times over the course of the study: Of the 11 dolphins, four (Dolly, Donna, Sunny, and Nami; see Table 1) were observed during each observation period. Jenny was absent from the group during the second observation (B). Three males (Arnie, Kai, and Noah) were only present during the first period (A) (Arnie and Kai; see Table 1) or the first and second periods (A,B) (Noah; see Table 1). All three were moved to other facilities in November 2016. Arnie and Kai were already separated from the group during the second observation period (B) for social management reasons. The oldest male, Moby, died of natural causes in September 2018 at approximately 58 years. One of the oldest females, Anke (see Table 1), died of natural causes in April 2020 at approximately 37 years of age. A new female (Nynke; see Table 1) arrived shortly after Moby passed away in October 2018.

As the three younger males (Arnie, Kai, and Noah) were monitored for only one or two observation periods (see Table 1), the behavioral data and trainer scoring were only analyzed for the other eight dolphins (see Table 1).

### 2.2. Behavioral Data Collection

**Figure 1:**
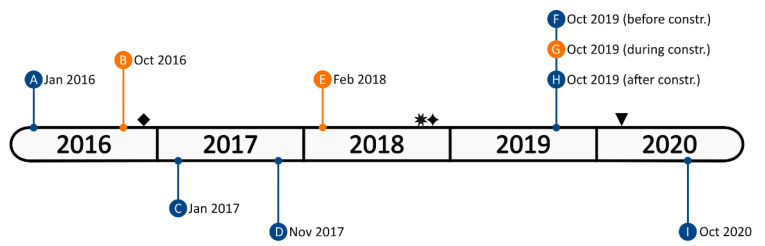
Timeline of all observation periods over the five-year span. All observations that were carried out during construction (B, E, and G) are highlighted in orange. ♦ Arnie, Kai, and Noah were transported to other facilities. ✸ Death of Moby. ✦ Arrival of Nynke. ▼ Death of Anke.

Observations were carried out over nine observation periods (see Figure 1) between January 2016 and October 2020. The observations were started regularly at least once per year or due to specific events, such as the arrival of a new animal, the death or departure (to another facility) of an animal, and during environmental disturbances, such as the construction work performed at the facility. During each observation period, data were usually collected on weekdays over at least 11 h, with a maximum of two sessions per day. If observations were conducted during construction periods (see Figure 1), the dolphins were monitored over the entire duration of the disturbance (B: 5 days; E: 8 days; G: 5 days).

Behavioral data were collected by two observers who were already familiar with the dolphins prior to collecting the data. First, an ethogram was compiled by using data from the literature and unpublished reports from previous studies carried out at the facility, as well as by conducting a preliminary two-week observational study using ad-libitum sampling [51]. Behavioral data were recorded using scan sampling (instantaneous sampling) [52] based on the ethogram, as shown in Table 2. The scan interval was defined as three minutes, as this time interval provided enough time to observe all of the animals in the group, and it has been regularly used in previous studies to measure the activity in bottlenose dolphins [53,54,55,56]. Each observation session lasted one hour. Observation sessions were always carried out between training, enrichment, or interaction sessions in the morning or in the afternoon. The individual overall activity budgets for each observation period were calculated by using instantaneous sampling data that were transformed into percentages. Additionally, the frequencies of the selected behavioral events (e.g., fast swimming, jumping, or regurgitation; see Table 3) were measured using all-occurrence sampling [52]. If all-occurrence behaviors that were also included in the scan sampling ethogram (agonistic and socio-sexual interactions) were recorded during scan sampling, these specific behavioral events were not included in the all-occurrence data to preclude pseudo-replication of these categories.

### 2.3. Training Performance

The dolphins’ performance during their daily training was evaluated by experienced trainers. Trainer-dolphin pairs could change from session to session and from day to day, due to different work schedules of the staff. The trainers only rated the animals with which they directly worked. The dolphins’ training performance was evaluated based on a three-point rating scale and calculated for each day that the dolphins were observed (Table 4). If a session was aborted by a trainer or a dolphin, this was recorded separately.

### 2.4. Statistical Analysis

Statistical analyses were performed with R, version 4.0.3 (R Core Team, 2020), using the RStudio interface (RStudio Team, 2021). Plots were generated with RStudio by using the *ggplot* [72] and *ggpubr* [73] packages. Individual differences between observation periods were tested with a Kruskal–Wallis rank-sum test if the data were normally distributed. If the data were not normally distributed, we applied a two-sided Wilcoxon signed rank test. The test for the normal distribution was performed with a Shapiro–Wilk normality test. To test the correlation between age and play/solitary behavior, we fitted a linear model. To see if fast swimming and the average trainer rating were different for different observation periods, we fitted a linear mixed model with fast swimming or the average trainer rating as a response variable, the observation period as a fixed factor, and the animal ID as a random factor. We used the lmer function of the *lme4* package [74]. We subsequently conducted Tukey’s post hoc test for pairwise comparisons of the observation periods by using the *emmeans* [75] package. We also compared periods with and without construction noise by using a two-sided Wilcoxon signed rank test. For this, we compared the mean values for each animal for the pooled observation periods with noise and pooled periods without noise. We also tested if the animals showed different proportions of affiliative, play, and solitary behavior for observation periods with noise compared to observation periods without noise. We used a linear mixed model with the proportions of the respective behaviors as response variables, the noise status as a fixed factor, and the animal ID as a random factor. For every parameter, we made a null model that excluded the fixed factor and included animal ID as a random intercept. We used ANOVAs to compare the null models to the models that included the respective fixed factor.

## 3. Results

### 3.1. Changes in Activity Budgets

**Figure 2:**
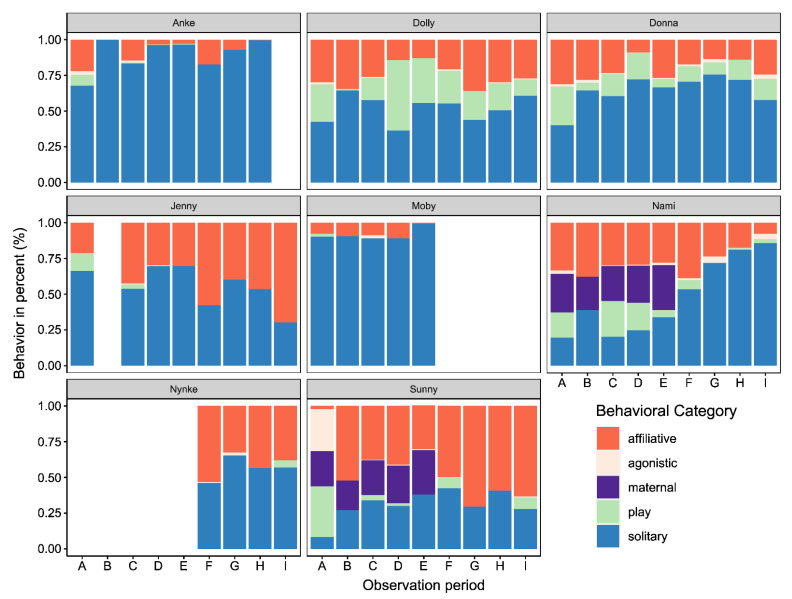
Activity budgets for each of the eight focal animals for five behavioral categories (see Legend).

The activity budgets for the eight focal animals are shown in Figure 2. During all the observation periods, Moby and Anke, the two oldest animals, showed more solitary behaviors than all of the other dolphins (see Figure 2; Wilcoxon rank-sum test, *p* < 0.05). For Moby, the proportions of solitary behavior and affiliative behavior did not differ across all observation periods until his death after observation period E (Kruskal–Wallis rank-sum test, p_sol_ = 0.79; p_aff_ = 0.24). For Anke, significant differences among the proportions of solitary behaviors across all observations were found (Kruskal–Wallis rank-sum test, p_sol_ < 0.001). Pairwise comparisons made by using Dunn’s test indicated solitary behavior during D and H to be significantly higher than those of the other periods (*p* < 0.05).

After Nynke arrived at the zoo, the proportion of affiliative behavior in Jenny’s activity budget increased (Wilcoxon signed rank-sum test, W = 1869.5, *p* < 0.0001). Nynke only showed affiliative and solitary behavior during the first three periods after she arrived (F–H). Nynke exhibited play behavior only on one day during the last observation period. The differences in the observed proportions of affiliative and solitary behavior were not significant (one-way ANOVA: *aff*: F_3,36_ = 0.912, *p* = 0.445; *sol*: F_3,36_ = 1.281, *p* = 0.296).

Dolly and Donna were the only two dolphins that were observed to play (see Figure 2) during all observation periods. However, the occurrence of play was impacted by construction noise. During the first observation period with construction noise (B), both dolphins showed less play behavior than during the first observations (A) (Wilcoxon signed rank-sum test, W_Dol_ = 97.5, W_Don_ = 94.5, *p* < 0.05). Donna also showed less play behavior during all observations with construction noise than during periods without (Wilcoxon signed rank-sum test, W = 1076.5, *p* < 0.05). Nami also demonstrated less play behavior during the periods with construction noise (Wilcoxon signed rank-sum test, W = 1131, *p* < 0.01). The other seven dolphins showed little to no play behavior.

The maternal behavior between Sunny and Nami was observed until observation period E (see Figure 2). After that (F–I), Sunny showed more affiliative behavior than before (A–E; Wilcoxon signed rank-sum test, W = 504.5, *p* < 0.0001). During observation period F, the proportion of affiliative behavior already increased (Wilcoxon signed rank-sum test, W = 19, *p* < 0.05). Nami’s activity budgets also changed after she was weaned at approximately 4.5 years of age. Although solitary behavior was not more frequent during observation period F (no disturbance; Wilcoxon signed rank-sum test, W = 29, *p* = 0.1023) than during period E (with disturbance), the proportion of solitary behavior increased from observation period D to F (no disturbance, Wilcoxon signed rank-sum test, W = 48.5, *p* < 0.05). Overall, the proportion of solitary behavior increased over all periods after weaning (A–E vs. F–I, Wilcoxon signed rank-sum test, W = 239.5, *p* < 0.001). Additionally, Nami showed less affiliative behavior (Wilcoxon signed rank-sum test, W = 1703, *p* < 0.01) and play behavior (Wilcoxon signed rank-sum test, W = 2033, *p* < 0.0001) after weaning (periods F–I). Although the activity budgets also showed an increase in agonistic interactions, the difference was not significant in comparison with all of the pre-weaning observations (Wilcoxon signed rank-sum test, W = 1121, *p* < 0.1027).

**Figure 3:**
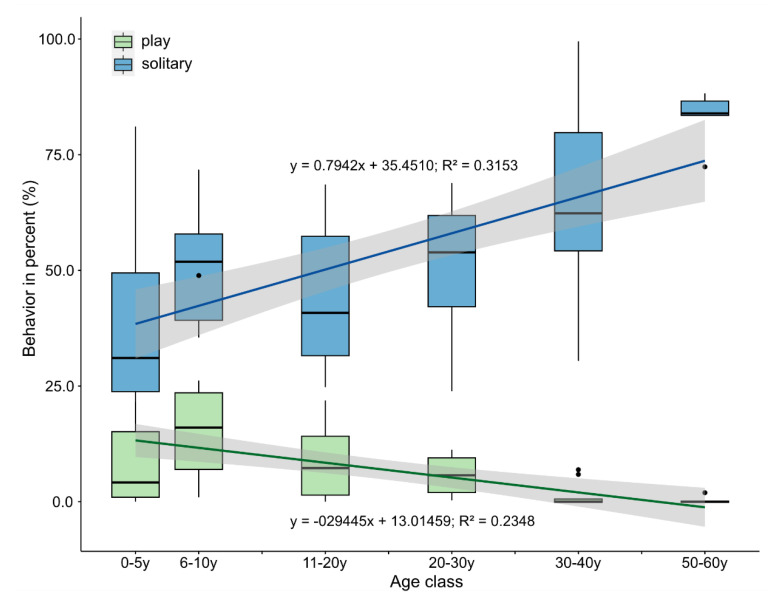
Age dependencies of play and solitary behavior. Play behavior decreased, and solitary behavior increased with increasing age. We used age categories to plot these dependencies.

We found that the ages of the animals affected the occurrence of play and solitary behavior. We fitted a linear regression model using data from all observed dolphins (including Arnie, Noah, and Kai; see Figure 3) and found that the proportion of solitary behavior increased with increasing age (R^2^ = 0.3153, F_1,63_ = 29.02; *p* < 0.0001). In addition, the proportion of play behavior decreased with increasing age of the dolphins (R^2^ = 0.2348, F_1,63_ = 19.33; *p* < 0.0001). We also calculated a linear regression model excluding all construction periods and found a similar correlation between increasing age and the frequency of play and solitary behavior, respectively (solitary behavior: R^2^ = 0.7630, F_1,42_ = 14.37; *p* < 0.001; play behavior: R^2^ = 0.36931, F_1,42_ = 19.06; *p* < 0.0001). We used age categories to plot the dependency on the age of play and solitary behavior (see Figure 3), while for the linear models, we used the absolute age.

### 3.2. Effects of Environmental Disturbances

#### 3.2.1. Affiliative, Play, and Solitary Behavior

The dolphins’ frequency of affiliative behavior did not differ between periods with construction noise and those without construction noise (linear mixed model (LMM), χ^2^(1) = 0.9254, *p* = 0.3361; data from all observations period with and from all periods without construction noise were pooled, respectively). Solitary behavior was also not affected by construction work (LMM, χ^2^(1) = 0.8441, *p* = 0.3582). However, play behavior changed, as the dolphins showed approximately 6.3% more play behavior during periods without construction noise than when construction was present (overall play rates: 21.72% during construction, 26.24% without construction; LMM, χ^2^(1) = 19.882, *p* < 0.0001).

#### 3.2.2. Fast Swimming

**Figure 4:**
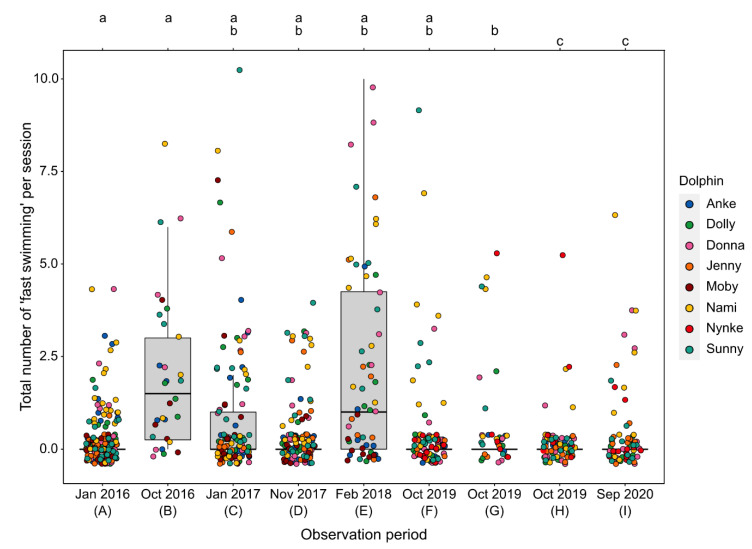
Frequency of fast swimming behavior for the different observation periods (**A**–**I**) observed for all eight focal dolphins (see Legend). Periods sharing a letter are not significantly different (based on Tukey’s post hoc test, *p* < 0.01).

**Figure 5:**
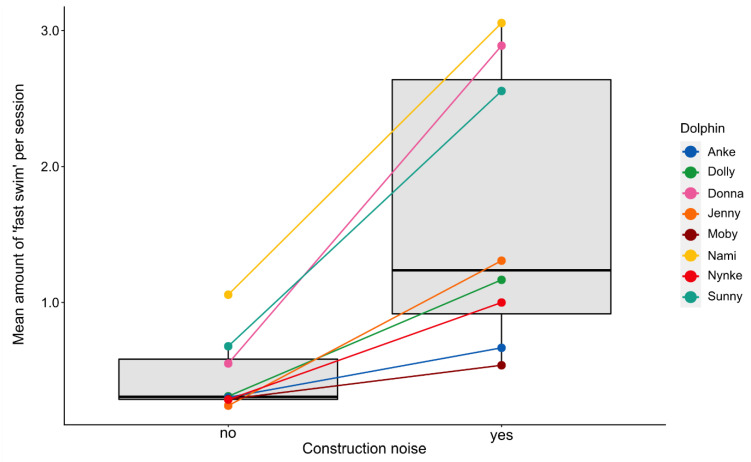
Mean frequencies of fast swimming observed during periods with and without construction noise. All periods with and all periods without construction noise were pooled to deduce a mean value for each individual.

The frequency of fast swimming differed significantly among the different observation periods (LMM, χ^2^(8) = 129.43, *p* < 0.0001, see Table 5). Using a pairwise post hoc comparison among all observation periods (see Figure S1, Supplementary Material), we found that, especially during the first (B) and second (E) construction periods, fast swimming significantly increased (see Figure 4). No significant differences in relation to construction noise were found between period G and the other periods without disturbances. However, for all dolphins, the mean frequency of fast swimming was significantly higher during periods with construction noise (pooled periods; Wilcoxon signed rank-sum test, *p* < 0.01; see Figure 5).

#### 3.2.3. Training Performance

**Figure 6:**
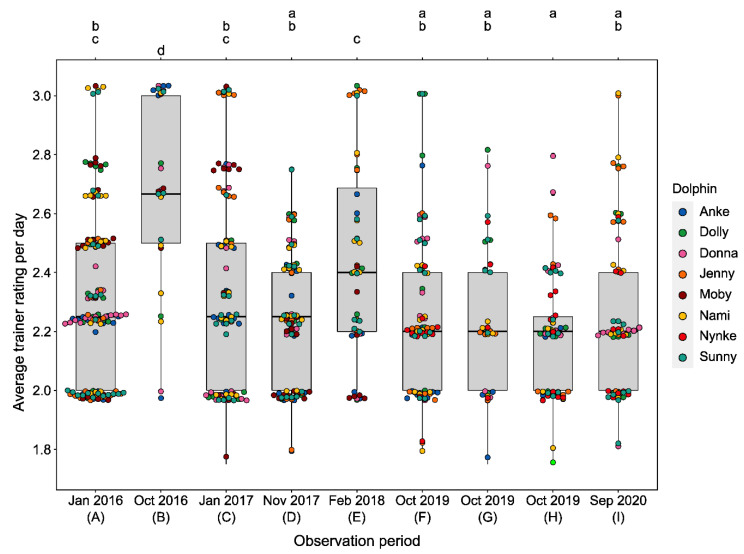
Differences in average training performance evaluated by the trainers after each session for all eight focal dolphins (see Legend) across all observation periods (**A**–**I**). Periods sharing a letter are not significantly different (based on Tukey’s post hoc test, *p* < 0.01).

**Figure 7:**
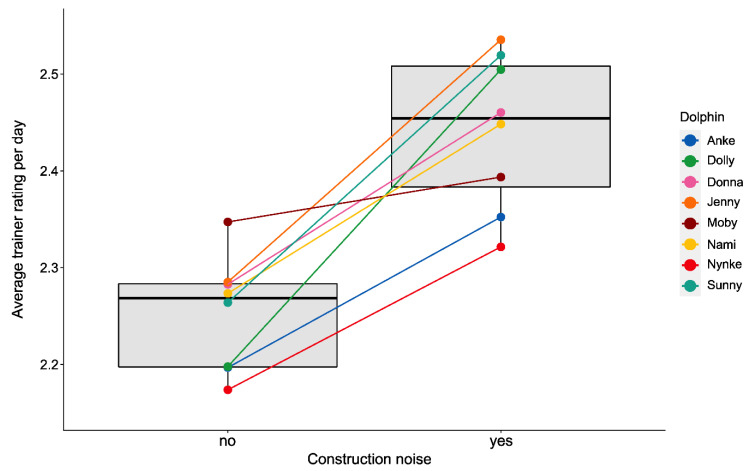
Mean differences in average trainer rating during periods with and without construction noise. All periods with and all periods without construction noise were pooled to deduce a mean value for each individual.

Similar to fast swimming, the average trainer rating differed among the different observation periods (LMM, χ^2^(8) = 104.9, *p* < 0.0001, see Table 5). The pairwise post hoc comparison (see Figure S2, Supplementary Material) showed that the dolphins’ average training performance significantly decreased (represented by higher average trainer ratings) during period B compared to all other periods (see Figure 6). In addition, the average training performance from observation period E was significantly lower than in most of the other observation periods (see Figure 6). Similar to the changes in the dolphins’ fast swimming behavior, their average training performance was significantly lower during the sessions with construction noise compared to the sessions that took place in times when there were no disturbances (Welch’s t-test, *p* < 0.001, see Figure 7). Moreover, on days with high frequencies of fast swimming (≥5 incidents per session), the dolphins’ average training performance was worse than on days when no fast swimming was observed (LMM, χ^2^(3) = 11.171, *p* < 0.05 see Figure S3, Supplementary Materials).

For periods with significant changes in the activity budgets, we investigated if the training performance also differed between these periods. During observation periods D and E, when Anke showed significantly more solitary behavior, no significant changes in her average training performance were found (Wilcoxon signed rank-sum test, *p* = 0.18). Post-weaning, Nami showed increased frequencies of solitary behavior. At the same time, her average training performance significantly decreased (Wilcoxon signed rank-sum test, W = 1876.5, *p* < 0.0001). Sunny’s average training performance did not change significantly after her calf (Nami) was weaned (Wilcoxon signed rank-sum test, W = 1408, *p* = 0.47). As already described, Jenny demonstrated higher levels of affiliative behavior after Nynke arrived in 2018. At the same time, her average training performance also improved (Wilcoxon signed rank-sum test, W = 888, *p* < 0.05).

## 4. Discussion

Our results show that behavioral studies are an important and sensitive tool to measure the impact of disturbances on dolphins under human care. The observed dolphins were monitored over a prolonged period of time in which not only external factors, but also life circumstances, e.g., the integration of new individuals, age, weaning processes, and deaths, resulted in changes within the group. These changes could be captured by using higher-order behavioral categories.

### 4.1. Activity Budgets

All dolphins showed distinct behavioral patterns. Solitary behavior, as well as affiliative behavior, were the most dominant behavioral categories shown by all dolphins (except for Sunny and Nami until observation period E). Aggressive and agonistic interactions, as well as sexual behavior, were observed the least. This finding is in line with those of previous studies that showed that females usually engage in less aggressive interactions than males, especially if a leading male is present [59,76]. While Moby was considered as the leading male of the group, he rarely interacted with the other animals, suggesting that his presence had only a small effect on the number of agonistic interactions among the other (female) dolphins. In addition, the absence of mature males might explain the low level of socio-sexual behavior observed, as socio-sexual interactions are most frequent in male–male dyads and between males and females [57,68].

We also found that the frequency of play and solitary behavior changed with increasing age of the dolphins. For Moby and Anke, the two oldest animals, the most observed behavior was solitary behavior. The lack of social interactions for Moby can be explained by the absence of other males within the group and the common occurrence of sexual segregation in bottlenose dolphins [77]. Male bottlenose dolphins form strong intraspecific bonds with other males [78,79], and mainly interact with females in estrous [58]. Thus, the absence of other males can, at least partly, explain Moby’s low level of social behavior.

Anke and Moby also displayed almost no play behavior. Moreover, we found that play behavior was negatively correlated with the age of the dolphin. This finding is comparable to those of other studies across different species [80]. For dolphins, it has already been shown that calves and juveniles play more than adults [65,81,82]. Here, only the youngest individuals (Dolly, Donna, and Nami) regularly exhibited play behaviors, including locomotory play, bubble ring, or objects play either during all periods (Dolly and Donna), or at least over five periods (Nami). In the other dolphins, play behavior was observed only sporadically. This is in contrast to the findings of a study by Walker et al. [23], where play behavior was one of the most frequently observed behaviors [23]. In addition, male dolphins tend to display more play behaviors than females [83,84]. Considering that our group mainly consisted of mature females, this could explain the low level of play behavior observed in the older animals. However, Dolly and Donna were the only ones who exhibited non-object play, such as bubble ring play, and also displayed play behaviors across all observation periods. Both dolphins were born at another facility (Zoo Duisburg, Germany) and were brought to Nuremberg Zoo in 2014. This particular play behavior is known to occur in the animals in Duisburg (Hüttner, personal observation). In general, the generation of such vortex rings is observed mainly in bottlenose dolphins, but Amazon River dolphins (*Inia geoffrensis*) and beluga whales (*Delphinapterus leucas*) have also been observed to play with vortex rings [81,85,86,87,88,89]. Dolphins generate vortex rings by using different techniques, such as heat strokes, fluke slapping, and expelling air underwater, and they visualize them by using self-controlled air injections [67]. Marten et al. [89] even described a “bubble ring culture” as a result of associative learning within a group of dolphins under human care. This would explain why all of the dolphins at Zoo Duisburg show this behavior. However, it is interesting that none of the Nuremberg dolphins copied this behavior. This implies that Dolly and Donna learned to play with air-filled bubble rings underwater prior to their arrival at Nuremberg Zoo, and therefore, showed play behavior more often than the other dolphins, which mainly played with human-made objects. While solitary play is considered an important behavior [83], its dependency on the provision of play or enrichment objects must be considered when interpreting these data. However, in our study, we did not record the presence of enrichment objects during the observation sessions or differentiate between the different forms of play.

Changes in the frequency of affiliative and solitary behavior are most likely associated with the dynamic group structures of dolphins [58,90,91]. Bottlenose dolphins live in so-called “fission–fusion” communities, which can change in group composition—in the wild, sometimes even on a daily basis [58]. After Nynke joined the group in September 2018, the frequency of affiliative behaviors in Jenny’s activity budgets significantly increased. A preliminary look at the individual interactions showed that Nynke only affiliated with Jenny, and vice-versa. However, a better understanding of the dolphins’ affiliation network is needed to understand these connections.

Two very clear and significant individual changes were observed in Sunny’s and Nami’s activity budgets as a result of Nami’s weaning between observation periods E and F. Typically, dolphin calves are nursed until four years of age [92,93]. In January 2016, when the observations began, Sunny and Nami engaged in specific mother–calf interactions for approximately 20% of their respective activity budgets. Such behavior was no longer observed in October 2019 (observations F–H).

Rather, Nami was mainly observed solitarily, while and play and affiliative behavior were almost nonexistent. Affiliative contacts, especially between conspecific animals of the same sex, are considered highly important for social species, such as the bottlenose dolphin, among other things, to maintain social bonds [41,57]. Thus, affiliative contacts have been identified as a positive welfare indicator in dolphins [36,42,43] and other zoo species [94]. Therefore, we suggest that low frequencies of affiliative interactions, such as synchronous swimming or flipper rubbing and at the same time a sudden increase of solitary behavior should, in turn, be considered as signs of a negative welfare state. However, this should still be viewed with caution, but will be further discussed below (see Section 4.2.2. for further details).

### 4.2. Influence of Environmental Disturbances and Welfare Implications

#### 4.2.1. Behavioral Changes

Several case studies have highlighted the importance of behavioral assessments during environmental disturbance periods for measuring how animals respond to these changes and evaluating how animal welfare is impacted at the individual level [27,95,96,97].

We found that all animals spent less time playing during construction periods than during periods without construction. Different studies have suggested that animals mainly engage in the display of playful behaviors in the absence of threats, stressors, or other challenges that potentially decrease their welfare [98,99]. Thus, play behavior has been identified as an easy and straightforward indicator for positive welfare or emotional state in non-human animals [98], including dolphins [46]. While, in our study, play behavior mainly included non-object and object play, the results are congruent with those of other studies that demonstrated that social play behavior diminished or disappeared when conditions became more stressful in a group of bottlenose dolphins under human care [46].

All dolphins exhibited increased fast swimming behavior during the construction periods, both at the group level and at the individual level. Especially during the first two phases with construction work (B and E), fast swimming activities were more frequent, whereas during the last construction period (G), no obvious increase was observed. Although habituation could be suggested, due to the time that elapsed between periods E and G, habituation was unlikely. Altogether, however, these results are comparable to previous findings. Serres et al. [24] observed similar results in different species under human care, including bottlenose dolphins, and showed that, in all species, fast swimming was observed more frequently in periods with disturbances. Studies of wild dolphins also demonstrated that fast swimming is a common stress response to disturbances, e.g., boat traffic, and also already showed that fast swimming activities in dolphins and other cetaceans increased during disturbances [24,100,101], especially if the disturbances were associated with an increase in air and/or underwater noise levels [102].

Other studies showed that socio-sexual behavior was negatively correlated with disturbances [44]. However, socio-sexual behavior was generally only recorded at very low rates, most likely due to the absence of a sexually active male or the all-female group after Moby passed away.

#### 4.2.2. Training Performance

The analysis of the keepers’ daily records of training performance and the calculation of the average trainer rating confirmed important assumptions based on our behavioral observations. Training performance, or Willingness to Participate (WtP), during daily training routines have already been shown to be valid—and especially early—indicators when it comes to evaluating an animal’s health or welfare state [31,40]. A recent study with bottlenose dolphins highlights that WtP even proved to be a more sensitive indicator compared to conservative measurements, such as daily medical examinations or food intake [19]. In our study, the average training performance and play behavior decreased, while the frequency of fast swimming increased during observation periods with construction noise. Additionally, on days with high rates of fast swimming, the dolphins’ training performance was also lower. These correlations underline our assumption that fast swimming should be used as an early and easy-to-observe measure to indicate stress in response to noise in dolphins. Analyzing the dolphins’ overall training performance lets us validate other changes in the dolphins’ activity budgets that could be linked to welfare. As already mentioned, Nami exhibited less affiliative and less play behavior and more solitary behavior after she was weaned. At the same time, Nami’s training performance also declined. While the increased frequency of solitary behavior together with a decrease of affiliative interactions already hinted at a possible welfare concern, due to social problems for Nami, analyzing her training performance confirmed this assumption. In addition, we found that Jenny engaged in significantly more affiliative contact after Nynke joined the group. This change in Jenny’s activity budget only suggested an increase in her overall welfare state. However, Jenny’s simultaneous improvement in training performance again verifies our finding.

## 5. Conclusions

Our findings demonstrate that activity budgets obtained via scan sampling, which are based on broad behavioral categories, such as affiliative, aggressive, playful, or solitary behavior, represent a valuable tool for assessing dolphin welfare. We could show that our method, combined with an assessment of certain key behaviors (behaviors with welfare implications, e.g., fast swimming), provides important information on the welfare state of dolphins, underlining the importance of behavioral observations as one of the key measures for animal welfare. The individual behavioral categories were influenced by the age of the dolphins, group composition, social structure, as well as external disturbances, such as construction noise. Although certain specific affiliative behaviors or play behavior are already considered a reliable positive welfare indicator in dolphins [43,44,46,82], we show that an increase or decrease of affiliative interactions in an animal’s activity budget could also reflect changes in an animal’s welfare state. More research should also be conducted on other groups of bottlenose dolphins using this approach, to confirm/verify our findings. As already proposed by Serres et al. [24], also our results validate fast swimming as an early and easy-to-measure indicator to assess potential stressful conditions in dolphins that need to be included in welfare assessments for dolphins.

Altogether, this study highlights the importance of systematic behavioral observations for welfare assessments with dolphins under human care. While focal sampling might provide more detailed activity budgets, time for collecting behavioral data is often limited at most facilities [103]. Instead, it might be more practical and applicable for the animal care staff to use behavioral data from instantaneous sampling to regularly monitor and evaluate dolphin welfare. We recommend conducting behavioral observations regularly—these serve as a behavioral baseline and provide information on the frequency of certain indicator behaviors (e.g., fast swimming) and how changes in the dolphins’ activity patterns can be attributed to external environmental changes or other factors. Thus, changes in the animal’s welfare state can be detected in time. Due to the manifold ways in which individuals respond and adapt to potential stressors, behavioral assessments should always be paired with additional measures, e.g., willingness to participate [40], measuring glucocorticoid metabolite levels, and monitoring daily food intake, or breathing rate [26], to create a holistic view of each individual’s wellbeing over time and during extraordinary events.

## Figures and Tables

**Table 1:** List of the individuals observed over the five-year span. The observation periods in which there was construction noise are highlighted in orange. Arnie, Kai, and Noah were not included in the dataset, if not stated otherwise, because they were observed for only one (Arnie and Kai) or two (Noah) observation periods.

Name	Sex	Date of Birth	Wild/Captive Born	Observation Period	Comments
Anke	F	approx. 1983	wild	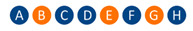	Died in April 2020
Arnie	M	18.06.2000	captive	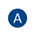	Transported to another facility in November 2016
Dolly	F	04.08.2007	captive	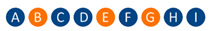	Half-sister to Donna
Donna	F	17.09.2007	captive	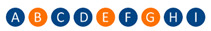	Half-sister to Dolly
Jenny	F	approx. 1987	wild	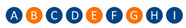	
Kai	M	21.08.2010	captive	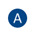	Transported to another facility in November 2016
Moby	M	approx. 1960	wild	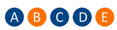	Died in September 2018
Nami	F	31.10.2014	captive	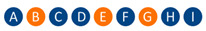	Calf of Sunny
Noah	M	16.11.1993	captive	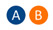	Transported to another facility in November 2016
Nynke	F	approx. 1983	wild	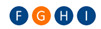	Transported to NBG in September 2018
Sunny	F	16.05.1999	captive	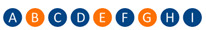	Mother to Nami

**Table 2:** Catalog of behavioral categories that were used during scan sampling. For each behavioral category, some examples are shown.

Category	Code	Definition	References
Affiliative	aff	All affiliative interactions between two or more dolphins, e.g., synchronous breathing, flipper rubbing, and pair swimming.	[41,57,58]
Agonistic	ago	Any form of agonistic or aggressive behavior, e.g., biting, (tail) slapping, ramming, or hitting (with rostrum or fluke).	[57,59]
Maternal	mat	Mother–calf interactions were defined as echelon swimming, nursing, or swimming in infant position.	[60,61,62]
Play	play	Object-play behavior, e.g., ball play, bubble ring play, locomotor play, or social play, between two or more dolphins.	[39,63,64,65,66,67]
Socio-sexual	sex	All behaviors between at least two dolphins with any form of body contact in the genital area, including goosing, petting, or rubbing of the genital area.	[57,68]
Solitary	sol	Behaviors shown by one dolphin without interactions with another individual, e.g., resting or swimming.	[57]

**Table 3:** All-occurrence behaviors, sampled during each observation.

All-Occurrence Behavior	Definition	References
Fast swimming	A dolphin swims at a higher speed than normal, indicated by powerful fluke strokes, riddles on its skin, or waves on the water’s surface.	[24]
Jumping	A dolphin performs an aerial behavior by leaping out of the water and diving headfirst.	[60]
Agonistic interaction	Any agonistic or aggressive behavioral events, e.g., biting, (tail) slapping, ramming, or hitting (with rostrum or fluke), between two or more individuals.	[57,69]
Socio-sexual interaction	All behaviors between at least two dolphins with any form of body contact in the genital area, including goosing, petting, or rubbing of the genital area.	[57,68]
Regurgitation	A dolphin regurgitates already swallowed fish at will. Regurgitation is indicated by the dolphin ejecting a plume of water and fish debris out of its mouth. Sometimes, whole fish are regurgitated and swallowed again.	[70,71]

**Table 4:** The three-point rating scale used by the trainers to evaluate each dolphin’s performance after each training session.

Rating	Dolphin Performance	Definition
1	excellent	The dolphin performed all of the conditioned behaviors correctly at a high level (above the average standard), showed a high level of motivation (e.g., returned to its trainer immediately), was highly focused, and took every fish reward without problems.
2	good	The dolphin performed all behaviors well, but with some minor mistakes, meeting the trainer’s expectations (e.g., conditioned behaviors are performed according to the established standard).
3	poor	The dolphin performed poorly, e.g., did not eat well, left the trainer, interacted with other dolphins, or performed most behaviors below the average standard, refused to perform certain behaviors, or was very distant from the trainer (no or only little eye contact).

**Table 5:** Statistical results of the Poisson distributed linear mixed-effect models (LMMs). The results show the effect of each observation period on the frequency of fast swimming, as well as the average overall trainer rating of each dolphin.

LMM-Model	Observation Periods	Estimate	Std. Error	Df	T-Value	*p*-Value
(a)						
Fast swimming	Intercept	0.36853	0.17284	18.18687	2.132	0.046873 *
Poisson	Observation period B	1.63546	0.27633	709.19429	5.918	<0.0001 ****
AIC: 2531.8	Observation period C	0.61732	0.17478	708.12697	3.532	0.000439 ***
Deviance: 2509.8	Observation period D	0.11255	0.17478	708.12697	0.644	0.519792
	Observation period E	1.97565	0.2155	708.12697	9.168	<0.0001 ****
	Observation period F	0.01046	0.18841	715.93166	0.056	0.955751
	Observation period G	0.22204	0.26123	714.31994	0.85	0.395621
	Observation period H	−0.29224	0.19627	715.9851	−1.489	0.136935
	Observation period I	0.03387	0.20829	715.6305	0.163	0.870888
(b)						
Average Trainer Rating						
Poisson	Intercept	2.31881	0.02379	64.29293	97.483	<0.0001 ****
AIC: 181.6	Observation period B	0.37637	0.05397	710.44062	6.974	<0.0001 ****
Deviance: 159.6	Observation period C	0.02191	0.03423	706.33135	0.64	0.52236
	Observation period D	−0.08809	0.03423	706.33135	−2.574	0.01027 *
	Observation period E	0.12611	0.04217	706.30673	2.991	0.00288 **
	Observation period F	−0.07889	0.03641	701.3391	−2.167	0.03058 *
	Observation period G	−0.06559	0.05076	713.89821	−1.292	0.19672
	Observation period H	−0.14983	0.03798	706.4147	−3.945	<0.0001 ****
	Observation period I	−0.04498	0.04015	690.11795	−1.12	0.26295

**** *p* < 0.0001, *** *p* < 0.001, ** *p* < 0.01, * *p* < 0.05.

## Data Availability

The data presented in this study are available on request from the corresponding authors. The data are not publicly available because they are owned by the owner of the animals and is only available with permission.

## Supplementary Materials

The following are available online at https://www.mdpi.com/article/10.3390/ani11072107/s1, Figure S1: Individual differences in fast swimming frequencies in between all periods with construction noise versus all periods without construction noise.; Figure S2: Individual differences in average training performance between all periods with construction noise versus all periods without construction noise.; Figure S3: Differences in average trainer rating in relation to the frequency of fast swimming.
