# Supplementary material for: Activity Budget Comparisons Using Long-Term Observations of a Group of Bottlenose Dolphins (Tursiops truncatus) under Human Care: Implications for Animal Welfare
Source: Animals (Basel). 2021 Jul 15;11(7):2107. doi: 10.3390/ani11072107 (PMC8300398; doi:10.3390/ani11072107)
Supplement: Supplementary file 1 [file animals-11-02107-s001.zip › animals-1255194-supplementary.pdf]

[1]Supplementary Material

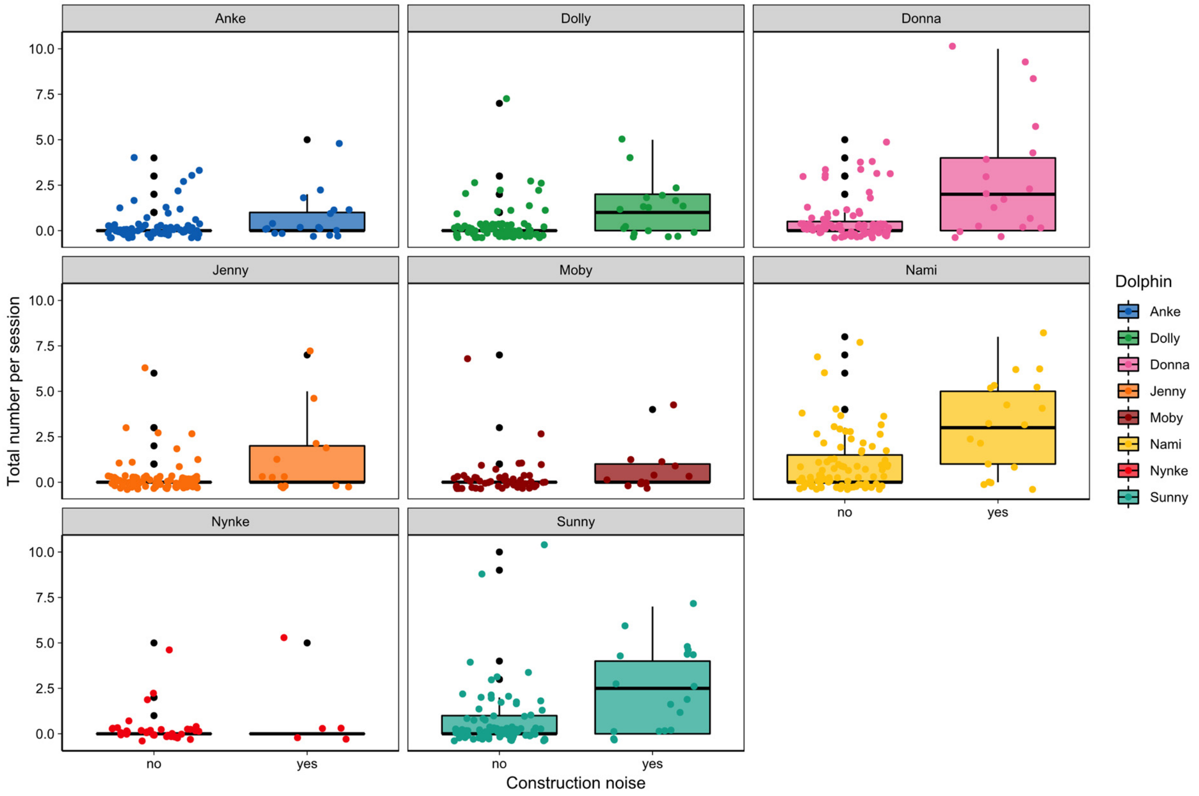

Figure S1. Individual differences in fast swimming frequencies in between all periods with construction noise versus all periods without construction noise. All dolphin exhibited increased more fast swimming during periods with construction work.

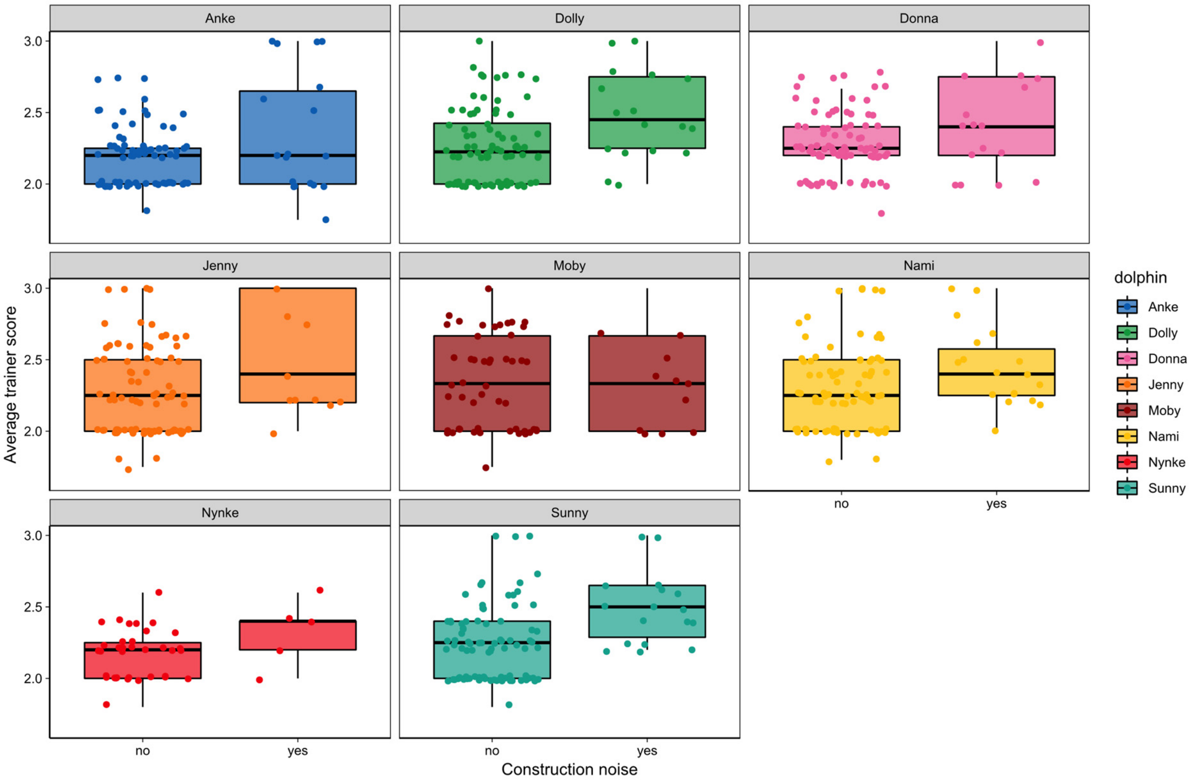

Figure S2. Individual differences in average training performance between all periods with construction noise versus all periods without construction noise. For all eight animals, training performance decreased during periods with construction work.

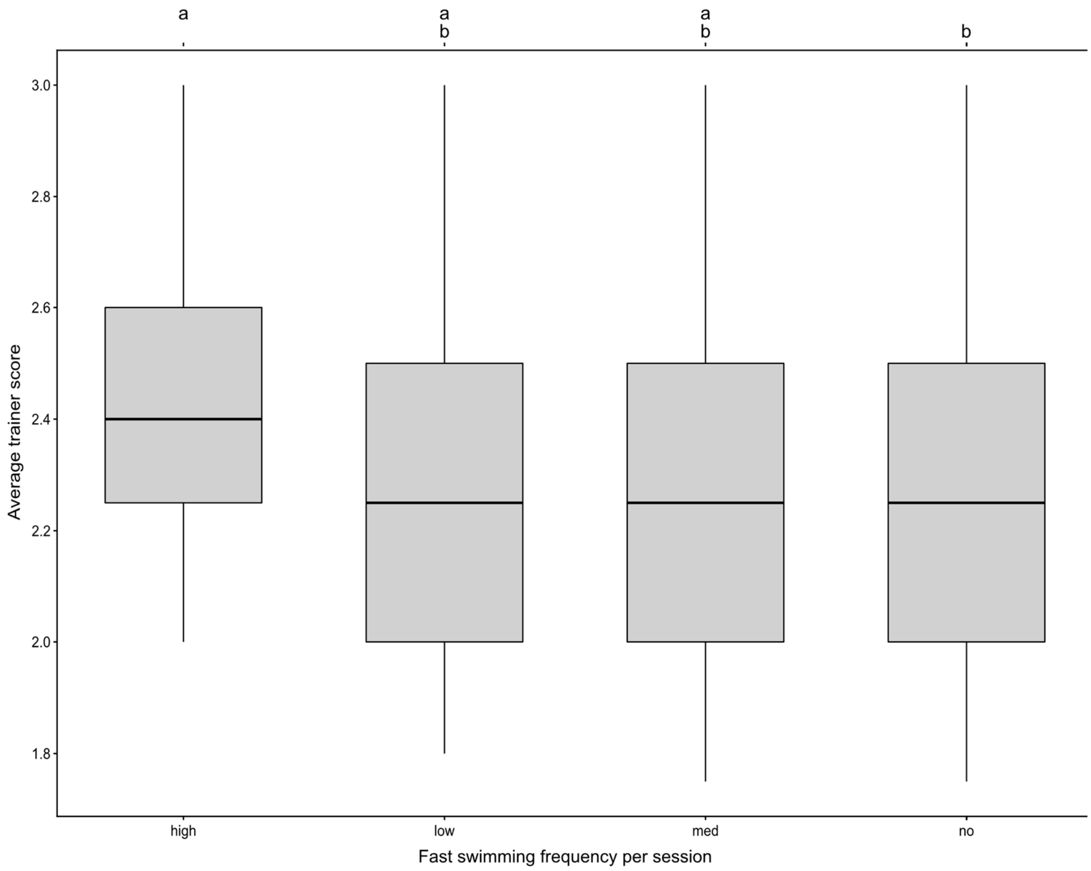

Figure S3. Differences in average trainer rating in relation to the frequency of fast swimming. On days with increased frequencies of fast swimming (high), the average trainer rating significantly decreased in comparison to days when no fast swimming was observed (no). Periods sharing a letter are not significantly different (based on Tukey's post hoc test, $p < 0.05$).

References

1. Wild, S.; Hoppitt, W.J.E.; Allen, S.J.; Krützen, M. Integrating Genetic, Environmental, and Social Networks to Reveal Transmission Pathways of a Dolphin Foraging Innovation. *Curr. Biol.* **2020**, doi:10.1016/j.cub.2020.05.069.
